# Current Clinical Trial Status and Future Prospects of PPAR-Targeted Drugs for Treating Nonalcoholic Fatty Liver Disease

**DOI:** 10.3390/biom13081264

**Published:** 2023-08-18

**Authors:** Shotaro Kamata, Akihiro Honda, Isao Ishii

**Affiliations:** Department of Health Chemistry, Showa Pharmaceutical University, Machida, Tokyo 194-8543, Japan; kamata@ac.shoyaku.ac.jp (S.K.); akihiro.honda.2103@gmail.com (A.H.)

**Keywords:** lanifibranor, saroglitazar, bezafibrate, pemafibrate, PPAR, dual/pan agonist, X-ray crystallography, NAFLD, NASH

## Abstract

The number of patients with nonalcoholic fatty liver disease (NAFLD)/nonalcoholic steatohepatitis (NASH) is increasing globally and is raising serious concerns regarding the increasing medical and economic burden incurred for their treatment. The progression of NASH to more severe conditions such as cirrhosis and hepatocellular carcinoma requires liver transplantation to avoid death. Therefore, therapeutic intervention is required in the NASH stage, although no therapeutic drugs are currently available for this. Several anti-NASH candidate drugs have been developed that enable treatment via the modulation of distinct signaling cascades and include a series of drugs targeting peroxisome proliferator-activated receptor (PPAR) subtypes (PPARα/δ/γ) that are considered to be attractive because they can regulate both systemic lipid metabolism and inflammation. Multiple PPAR dual/pan agonists have been developed but only a few of them have been evaluated in clinical trials for NAFLD/NASH. Herein, we review the current clinical trial status and future prospects of PPAR-targeted drugs for treating NAFLD/NASH. In addition, we summarize our recent findings on the binding modes and the potencies/efficacies of several candidate PPAR dual/pan agonists to estimate their therapeutic potentials against NASH. Considering that the development of numerous PPAR dual/pan agonists has been abandoned because of their serious side effects, we also propose a repositioning of the already approved, safety-proven PPAR-targeted drugs against NAFLD/NASH.

## 1. Introduction

Countermeasures against drastic increases in the numbers of patients with nonalcoholic fatty liver disease (NAFLD) caused by the global increase in the obese population have become an extremely urgent issue [1]. NAFLD is defined by hepatic steatosis that is not caused by significant alcohol consumption, the use of a steatogenic medication, or monogenic hereditary disorders [2]. A recent meta-analysis reported that the prevalence of NAFLD worldwide had increased significantly over time, from 25.5% in or before 2005 to 37.8% in 2016 or later [3]. Most forms of NAFLD include benign fatty liver called nonalcoholic fatty liver (NAFL). The remaining 20% of NAFLD cases progress to nonalcoholic steatohepatitis (NASH) [4] that is defined by diagnoses of steatosis, hepatocyte ballooning, and inflammation with or without fibrosis [2]. An estimated 20% of patients with NASH will develop cirrhosis (the formation in the liver of scar tissue known as fibrosis), which can cause the onset of hepatocellular carcinoma (HCC) [5]. NASH can return to NAFL or a normal liver, either spontaneously or through lifestyle modification; however, no cure is currently available for cirrhosis, and the only treatment available is to slow the progression or to perform a liver transplant [6].

Currently, no effective recommended drugs are available for treating NASH, and treatments such as pioglitazone (a thiazolidinedione (TZD; also called glitazone)-class drug), vitamin E medications (to reduce oxidative stress), and bariatric surgery are only available for symptomatic conditions [2]. To change this situation, various NAFLD/NASH drugs with different sites of action, including farnesoid X receptor agonists, thyroid hormone receptor agonists, C-C chemokine receptor 2/5 inhibitors, apoptosis signal-regulating kinase 1 inhibitors, and galectin-3 inhibitors, have been developed [7,8]. However, many drugs have already been withdrawn because of their serious side effects or insufficient or lack of therapeutic effects, and only some remain in the current clinical trials. Among them are a series of drugs targeting the nuclear receptor-type transcriptional factors and peroxisome proliferator-activated receptors (PPARs) that have three cognate subtypes (PPARα, PPARδ(/β), PPARγ), and these appear to among the most promising drugs for the treatment of NAFLD/NASH. PPARα regulates lipid metabolism mainly in the liver and skeletal muscle and glucose homeostasis via direct transcriptional control of the genes involved in peroxisomal/mitochondrial β-oxidation, fatty acid uptake, and triglyceride catabolism, and PPARα agonists, including fibrates, are used to treat hypertriglyceridemia. PPARγ is most highly expressed in white/brown adipose tissues, where it acts as a master regulator of adipogenesis and a potent modulator of whole-body lipid metabolism and insulin sensitivity. PPARγ agonists, such as TZDs, are used for type 2 diabetes mellitus (T2DM) [9]. PPARδ is ubiquitously expressed and controls energy metabolism and cell survival. PPARδ agonists are not yet clinically available but are expected to treat metabolic or cardiovascular diseases. Only a PPARδ-selective agonist (seladelpar) is in a clinical trial for treating primary biliary cholangitis (PBC) [10]. Notably, PPAR dual agonists (that act on any two of three subtypes) and PPAR pan agonists (that act on all three subtypes) are more expected than PPAR subtype-selective (specific) agonists for treating NAFLD/NASH. Here, we review the current status of clinical trials and discuss the prospects of new therapy for NAFLD/NASH, partly based on our recent functional and structural findings of several PPAR agonists [11,12,13,14].

## 2. PPAR Agonists in the Past and Current Clinical Trials against NASH

The United States (US) National Institutes of Health’s Clinical Trials Information website (https://clinicaltrials.gov/; accessed on 14 August 2023) contained the details of 42 clinical trials for NAFLD/NASH using nine PPAR agonists that had been so far completed or terminated (Table 1). In initial phase 2a trials, only the therapeutic effects (i.e., improvement of hepatic steatosis and fibrosis) were evaluated with noninvasive liver tests, such as transient, shear wave, acoustic radiation, magnetic resonance elastography, and serum scores such as aspartate aminotransferase (AST) to alanine aminotransferase (ALT) ratios, AST to platelet ratio index (APRI), Fibrosis-4 (FIB-4), and NAFLD fibrosis scores. In 2019, the US Food and Drug Administration (FDA) recommended that phase 2b trials should be accessed by liver biopsy to detect evidence of efficacy on a histological endpoint (i.e., reduction in inflammatory changes, improvement in fibrosis, or both) [7]. Table 2 lists the current ongoing 19 clinical trials for NAFLD/NASH that involve the use of chiglitazar, lanifibranor, saroglitazar, and pioglitazone.

We recently used X-ray crystallography to reveal the cocrystal structures of the PPARα/δ/γ-ligand-binding domain (LBD)–various ligands and registered all data in the PDB (63, 54, and 288, respectively, until 2023/8/16; among which 37 (58%), 4 (7.4%), and 6 (2.1%) registrations were derived from our laboratory), demonstrating their extremely diverse binding modes to relatively large ligand-binding pockets (LBPs) [11,12,13,14]. We also analyzed the ligand-induced PPARα/δ/γ activation status using two different methods: the GAL4-based transactivation assay and time-resolved fluorescence energy transfer (TR-FRET)-based PGC1α coactivator recruitment assay [11,12,13,14]. The transactivation assay detects the yeast GAL4-human PPARα/δ/γ-LBD chimera-dependent gene transcription activation in Cos-7 cells where the potentially confounding effects of endogenous PPARs are eliminated. Ligand binding to PPARα/δ/γ-LBD induces the dissociation of corepressor protein(s) and the association of the coactivator protein(s) complexes for linking to the basal transcriptional machinery. The PGC1α recruitment assay detects the association of the PGC1α coactivator to PPARα/δ/γ-LBD by highly sensitive TR-FRET. In those assays, both potencies, as evaluated by the half maximal effective concentration (EC_50_), and efficacies, as assessed by percent responses of the maximal response by the full (selective) agonist (GW7647 for PPARα, GW501516 for PPARδ, and GW1929 for PPARγ), were calculated [11,13]. Therefore, we have added the structural and functional information of several candidate PPAR agonists for a better understanding of their pharmacological/therapeutic effects.

### 2.1. Lanifibranor (PPAR Pan Agonist)—Under Consideration for Treating NAFLD/NASH

**Figure 1:**
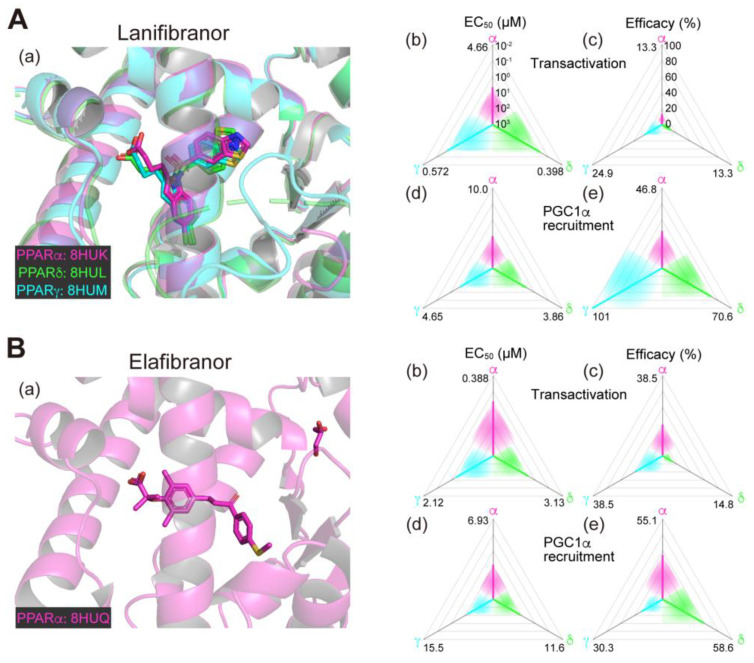

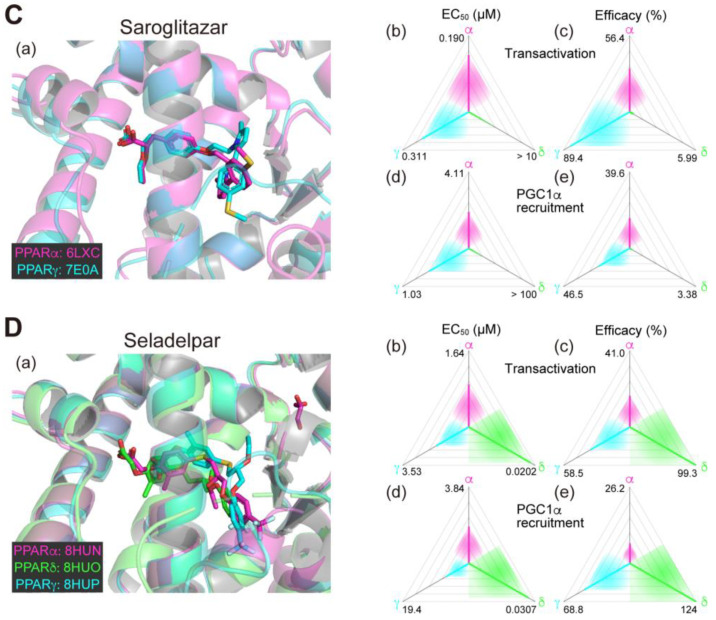
Binding modes in the PPAR cocrystal structures and the potencies/efficacies in transactivation and PGC1α recruitment activity of lanifibranor (**A**), elafibranor (**B**), saroglitazar (**C**), and seladelpar (**D**) against PPARα/δ/γ. (**a**) Merged magnified views of ligands bound to the PPARα (magenta)/δ (green)/γ (light blue)-ligand binding domains revealed by X-ray diffraction analyses of cocrystals; Protein Data Bank (PDB) IDs are shown. PPARδ/γ–elafibranor and PPARδ–saroglitazar cocrystals were not obtained. (**b**–**e**) Potencies as EC_50_ values (µM) (**b**,**d**), and efficacies as % of the maximal responses triggered by the PPARα/δ/γ-selective full agonists (GW7647, GW501516, and GW1929, respectively) (**c**,**e**) in GAL4-based transactivation assay in Cos-7 cells (**b**,**c**) and time-resolved fluorescence energy transfer (TR-FRET)-based PGC1α coactivator recruitment assay (**d**,**e**). In each ternary plot, the degrees of potency and efficacy are shown by the axes from the triangle center to the three vertices (PPARα in magenta, PPARδ in green, and PPARγ in light blue) on logarithmic (**b**,**d**) and linear scales (**c**,**e**), respectively. All structural and functional data were published by our group [11,12,13,14,20].

Lanifibranor (IVA337), developed by Inventiva Pharma (Daix, France), is a non-TZD/non-fibrate PPAR agonist that can activate all PPARα/δ/γ subtypes with “well-balanced” potencies/efficacies [15,16]. In our analyses, lanifibranor bound to very similar positions in PPARα/δ/γ-LBD (Figure 1A(a)) and activated PPARα/δ/γ-LBD with similar potencies and efficacies in both functional assays (Figure 1A(b–e)) [11,12,13,14]. In preclinical mouse experiments led by Inventiva, lanifibranor effectively prevented liver steatosis, inflammation, ballooning, and fibrosis [15]. In a phase 2b trial involving 247 patients with highly active NASH (NCT03008070; Table 1), the percentage of patients who had a decrease of at least 2 points in the Activity part of the Steatosis, Activity, and Fibrosis score (the scoring system that incorporates scores for ballooning and inflammation) without worsening of fibrosis was significantly higher with a 1200 mg/day lanifibranor dose than with placebo [17,18]. In an ongoing phase 3 trial for NASH in the US and other countries (NCT04849728; Table 2), 1000 patients will be randomly assigned to receive 800 or 1200 mg/day lanifibranor or matching placebo to investigate the resolution of NASH and the improvement of fibrosis as the primary endpoints.

### 2.2. Chiglitazar (PPAR Pan Agonist)—Under Consideration in China

Chiglitazar, a non-TZD PPAR pan agonist, was developed by Chipscreen Biosciences (Guangdong, China). This reagent is not commercially available, and we were unable to obtain its pharmacological and cocrystal structure information. Chiglitazar was approved in China in October 2021 for treating T2DM and NASH [19]. In a phase 2 clinical trial for NASH in China (NCT05193916; Table 2), 100 patients were randomly assigned to receive chiglitazar at 48 or 64 mg daily or a placebo with the liver fat content after an 18-week treatment as the primary endpoint and its results have not yet been posted.

### 2.3. Elafibranor (PPARα/δ Dual Agonist)—Discontinued

Elafibranor (GFT505), developed by GENFIT (Loos, France), was the first PPAR dual (/pan) agonist to treat NASH that was evaluated in clinical trials. In our analyses, the cocrystals with elafibranor were only obtained with PPARα-LBD (Figure 1B(a)), although this can activate all PPARα/δ/γ-LBDs with similar potencies (Figure 1B(b–e)) [13]. The reason why PPARδ/γ-LBD cocrystals could not be obtained despite the use of several crystallization methods [14,20] is unknown, but our heat stability analyses using circular dichroism (CD) revealed that elafibranor is an exceptional PPAR ligand in that its binding to PPARα/δ/γ-LBD did not stabilize its active (α-helical) conformation [13]. Supported by several positive results in animal experiments [21,22], elafibranor entered into clinical trials. In the phase 2 clinical trial (NCT01694849; Table 1), the efficacy and the safety of elafibranor at 80 and 120 mg/day for 52 weeks were evaluated in 275 patients with NASH. Although significant differences were absent in the primary endpoint that was defined as the proportion of patients with resolution of NASH and without fibrosis progression, the new NASH scoring system (proposed at the end of the study) did reveal a significant therapeutic effect [23]. The subsequent phase 3 clinical trial (NCT02704403; Table 1) enrolled 2157 participants, mainly from the US and Europe. The interim analysis in May 2020 showed that the safety and the tolerability were consistent with previous studies but elafibranor did not have a significant effect on the primary endpoint of resolution of NASH without worsening fibrosis [24]. Consequently, the clinical trial of elafibranor for NASH was discontinued in March 2022.

### 2.4. Saroglitazar (PPARα/γ Dual Agonist)—Under Consideration

Saroglitazar is the first glitazar developed by Zydus Therapeutics (Gujarat, India) to be granted for marketing authorization in India for treating diabetic dyslipidemia with its potent PPARα and moderate PPARγ activities [25]. Saroglitazar was then approved in India as an anti-NASH therapeutic in March 2020 but has not been approved in other countries. In our analyses, saroglitazar bound to and activated PPARα/γ-LBD but not PPARδ-LBD because of a steric hindrance (Figure 1C(a–e)) [12,14]; therefore, it is considered as a rare approved PPARα/γ dual agonist. No open research regarding the use of saroglitazar in animal experiments has been reported, although several clinical observational studies and case reports have been described from India [26,27,28]. In the phase 2 clinical trial (NCT03061721; Table 1) involving 106 NAFLD/NASH patients in the US, saroglitazar (1, 2, and 4 mg/day) and placebo were applied for 16 weeks. Saroglitazar (4 mg/day) significantly improved blood ALT levels (the primary endpoint), and the hepatic fat content, insulin resistance, and atherogenic dyslipidemia (the secondary endpoints) [29]. The phase 2b clinical trial (NCT05011305; Table 2) is currently recruiting US participants with the primary endpoint of resolution of NASH without worsening fibrosis after 76 weeks of treatment with 2 and 4 mg/day doses. Although major adverse events have not been reported in any clinical trial performed in India, careful attention should be paid to whether saroglitazar improves NASH or not and to what extent the adverse events are compared with those observed with other (abandoned) PPARα/γ dual agonists.

### 2.5. Seladelpar (PPARδ-Selective Agonist)—Interrupted

Seladelpar (MBX-8025), developed by CymaBay Therapeutics (Newark, CA, USA), is a rare PPARδ-selective drug. We found that seladelpar bound to all PPARα/δ/γ-LBDs (Figure 1D(a)) and activated all PPARα/δ/γ subtypes, but the EC_50_ values in both biological assays were 2–3-fold lower in PPARδ than in PPARα/γ (Figure 1D(b–e)) [13]. A phase 2 clinical trial (NCT03551522; Table 1) had been initiated with 181 patients with NASH in June 2018, but it was interrupted in November 2019 because of atypical histological findings, including histology characterized as an interface hepatitis presentation, with or without biliary injury, in patients who demonstrated improvement or stabilization of their biochemical measures of inflammation and liver injury and no liver-related adverse events after a 52-week treatment [10]. However, a subsequent in-depth investigation by an independent expert review panel (involving world-renowned liver pathologists and histologists) concluded that there was no clinical, biochemical, or histological evidence of seladelpar-related liver injury in the study and unanimously supported re-initiating the clinical development of seladelpar, and thus, the FDA lifted clinical holds on seladelpar in July 2020 [30]. The clinical trial for NASH has not resumed since then.

### 2.6. Fenofibrate (PPARα(/γ Dual) Agonist)—Discontinued

**Figure 2:**
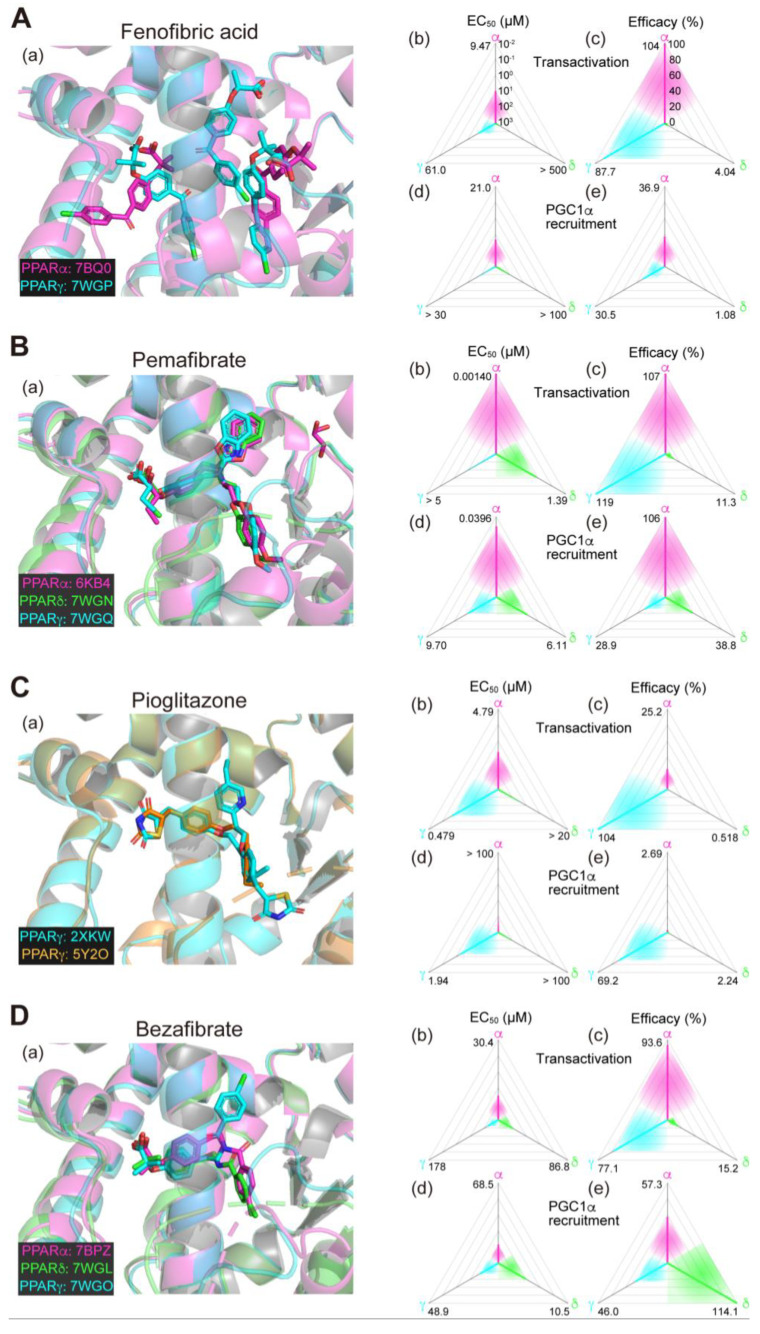
Binding modes in the PPAR cocrystal structures and the potencies/efficacies in transactivation and PGC1α recruitment activity of fenofibric acid (the active metabolite of fenofibrate) (**A**), pemafibrate (**B**), pioglitazone (**C**), and bezafibrate (**D**) against PPARα/δ/γ. (**a**) Merged magnified views of ligands bound to the PPARα (magenta)/δ (green)/γ (light blue)-ligand binding domain revealed by X-ray diffraction analyses of cocrystals; PDB IDs are shown. PPARδ–fenofibric acid and PPARα/δ–pioglitazone cocrystals were not obtained. (**b**–**e**) Potencies as EC_50_ values (µM) (**b**,**d**) and efficacies as % of the maximal responses triggered by the PPARα/δ/γ–selective full agonists (GW7647, GW501516, and GW1929, respectively) (**c**,**e**) in GAL4-based transactivation assay in Cos-7 cells (**b**,**c**) and TR-FRET-based PGC1α coactivator recruitment assays (**d**,**e**). In each ternary plot, the degrees of potency and efficacy are shown by the axes from the triangle center to the three vertices (PPARα in magenta, PPARδ in green, and PPARγ in light blue) on logarithmic (**b**,**d**) and linear scales (**c**,**e**), respectively. All structural (except for Figure 2C(a)) and functional data were published by our group [11,12,13,14,20].

Fenofibrate is a widely used fibrate developed by Groupe Fournier SA of France as a hyperlipidemic (triglyceride-lowering) agent that has a relatively low selectivity for PPARα. In our analyses, two and three molecules of fenofibric acid (an active metabolite of fenofibrate) bound to PPARα-LBD and PPARγ-LBD, respectively (Figure 2A(a)), and activated both PPARα/γ-LBD but not PPARδ-LBD with some preference for PPARα-LBD (Figure 2A(b–e)) [11,14]. Six clinical trials using fibrate for NAFLD/NASH have been conducted but none of them demonstrated significant therapeutic effects (Table 1). In a double-blind, randomized, placebo-controlled study (NCT02354976), fenofibrate decreased serum triglyceride levels but increased the total liver and liver fat volumes, indicating the complex nature of its pharmacological effects [31]. No new trials have been initiated since then.

### 2.7. Pemafibrate (PPARα-Selective Agonist)—Under Consideration in Japan

Pemafibrate (K-877), recently developed by Kowa Company (Nagoya, Japan), is classified as a selective PPARα modulator (SPPARMα) for its high PPARα selectivity and efficacy [31]. Pemafibrate potently decreases blood triglyceride levels and increases HDL-cholesterol levels at doses as low as 0.2 mg/day [32]. Pemafibrate is mainly metabolized by the liver with little excreted into the urine, whereas other fibrates such as fenofibrate and bezafibrate are mainly metabolized by the kidney and can therefore be used in diabetic patients with mild renal impairment [33]. In our analyses, pemafibrate bound to similar positions with its Y-shaped structure that fits into the PPARα/δ/γ-LBP (Figure 2B(a)). Pemafibrate only activated PPARα at lower doses but did activate PPARγ and, at much lesser extents, PPARδ at higher doses (Figure 2B(b–e)) [11,14]. Therapeutic doses of pemafibrate are very low (0.2 mg/day recommended and 0.4 mg/day at maximum) and it only activates PPARα at those therapeutic doses. In different mouse NASH models, pemafibrate significantly improved NASH conditions such as hepatic inflammation/fibrosis, ballooning degeneration, and biochemical scores [33,34]. A phase 2 clinical trial (NCT03350165; Table 1) involving 118 patients with NASH in Japan was completed in April 2021 [35]. Pemafibrate did not change the primary efficacy endpoint of the magnetic resonance imaging proton density fat fraction (MRI-PDFF) in the liver, but did significantly reduce liver stiffness as evaluated using magnetic resonance elastography without affecting safety endpoints (incidence of adverse events and adverse drug reactions after the drug administration). Overall, pemafibrate is considered a promising treatment candidate for NAFLD/NASH as well as a potential candidate for combination therapy with statins to treat atherogenic dyslipidemia [36,37,38].

### 2.8. Pioglitazone (PPARγ-Selective Agonist)—Under Consideration

Pioglitazone is a TZD-class T2DM drug developed by Takeda Pharmaceuticals (Osaka, Japan) that decreases blood glucose levels by improving insulin resistance in the skeletal muscle and the liver [39]. Two cocrystal structures of PPARγ-LBD–pioglitazone have been reported so far: PDB IDs 2XKW (not yet published) and 5Y2O [40]. Two incompatible (*R*)-pioglitazone binding modes are present in the former structure but only a single (*S*)-pioglitazone binding mode (that matches one of the two modes in the former) is present in the latter cocrystals (Figure 2C(a)). In our functional analyses, pioglitazone activated PPARγ and then PPARα at much lesser efficacies (Figure 2C(b–e)) [13] and we have so far failed to obtain a PPARα–pioglitazone cocrystal probably because of the low affinity against PPARα. Thus, pioglitazone is actually considered a PPARγ-selective agonist. Although four clinical trials have been conducted using pioglitazone to treat patients with NAFLD who also have T2DM (Table 2), and its efficacy in insulin-resistant NASH has been recognized in Japanese guidelines, its use in treating patients with NAFLD who did not have T2DM has been limited [41]. In the practice guidance from the American Association for the Study of Liver Diseases, pioglitazone is recommended for patients with or without T2DM with biopsy-proven NASH to improve liver histology, although it should not be used without biopsy-proven NASH because of its safety issues [2]. A phase 3 clinical trial (NCT00063622; Table 1) involving 247 nondiabetic patients with NASH indicated that pioglitazone (30 mg/day) for 96 weeks was effective even though weight gain was an adverse effect [42]. A phase 2 clinical trial (NCT01068444; Table 1) on 90 patients with NASH demonstrated the efficacy of a 24-week course of pioglitazone (30 mg/day) in reducing lipidosis and improving the inflammation and histology of NASH without worsening fibrosis [43]. In an ongoing phase 2b trial using pioglitazone (NCT04501406; Table 2), the primary endpoint is set as an improvement of ≥2 points in the NAFLD activity score without an increase in fibrosis stage. However, it has not yet become the first-line drug for nondiabetic patients with NASH and its main purpose is to improve diabetic symptoms in patients with NASH. When pioglitazone and other TZDs are administered for an extended period, attention should be paid for severe adverse effects, such as weight gain, heart failure, and the risk of bone fractures, caused by prolonged PPARγ activation. In animal experiments, PXL065 (deuterium-stabilized (*R*)-pioglitazone) exerted its therapeutic effect on NASH without causing weight gain or fluid retention, probably through nongenomic actions [44], irrespective of whether the second binding mode is involved or not. Since all TZDs currently in use are stereoisomeric mixtures, the development of new drugs by isomer stabilization is also expected.

### 2.9. Rosiglitazone (PPARγ-Selective Agonist)—Discontinued

Rosiglitazone was developed by GlaxoSmithKline and approved in the US and Europe (in 1999 and 2000, respectively) and had been widely used to treat patients with T2DM. However, a meta-analysis in 2007 reported serious concerns about its risk of cardiac injury and its use has been discontinued in many countries since then [45,46]. In a previous phase 2 clinical trial (NCT00492700; Table 1) on 63 patients with NASH, rosiglitazone (8 mg/day) taken for one year reduced blood ALT levels and improved fatty degeneration, but had no significant effects on liver fibrosis or NASH activity scores [47]. Subsequently, no benefits were found (beyond the risks) in improving insulin resistance and NASH conditions, even after an additional two-year extension of the treatment period [48].

### 2.10. Lobeglitazone (PPARγ Agonist)—Under Consideration in Korea

Lobeglitazone is a TZD that was developed by Chong Kun Dang Pharmaceutical Corp. (Seoul, South Korea) and approved for the management of T2DM in Korea in 2013 [49]. A phase 4 clinical trial (NCT02285205; Table 1) revealed that treatment with lobeglitazone reduced intrahepatic fat content as assessed by transient liver elastography, and improved glycemic, liver, and lipid profiles in patients with T2DM and NAFLD [50]. Further clinical trials are awaited for a favorable safety profile.

## 3. Future Prospects of PPAR Agonists for NAFLD/NASH

Numerous PPAR dual/pan agonists have been developed to treat T2DM, cardiovascular diseases, dyslipidemia, obesity, hypertension, PBC, renal dysfunctions, neurological diseases, psychiatric disorders, autoimmune diseases, inflammatory diseases, malignancies, and NAFLD/NASH [9]. Such PPAR dual/pan agonists combine the beneficial effects of PPAR selective agonists and may counteract inflammation and NASH progression more potently [51]. However, the development of several PPARα/γ agonists (e.g., naveglitazar, tesaglitazar, aleglitazar, ragaglitazar, peliglitazar, reglitazar, cevoglitazar, farglitazar, muraglitazar, imiglitazar, and sipoglitazar) and PPAR pan agonists (sodeglitazar and indeglitazar) have been discontinued in both preclinical and clinical studies, mainly because of cardiovascular adverse events, congestive heart failures, and the subsequent increased mortality perhaps caused by their full (potent) PPARγ activation [9]. Meanwhile, activation of PPARα may not cause such serious adverse effects because the full agonist pemafibrate has been safely used so far in Japan, and the evaluation of the adverse effects by PPARδ activation requires further clinical trials using PPARδ-selective agonists such as seladelpar.

The PPAR pan agonist lanifibranor is located on the front line of drug approval for NASH/NAFLD. The well-balanced features (in PPARα/δ/γ selectivity; Figure 1A(b–e)) could be underscored by the similar binding modes to the PPARα/δ/γ-LBD where the carboxylic group of lanifibranor bound to the four surrounding consensus amino acids (Ser280, Tyr314, His440, and Tyr464 in PPARα, Thr253, His287, His413, and Tyr437 in PPARδ, and Ser289, His323, His449, and Tyr473 in PPARγ) via hydrogen bonds and electrostatic interactions (Figure 1A(a)) to stabilize Activation Function-2 (AF-2) helix 12 for coactivator recruitment/PPAR activation [13]. The EC_50_ values of lanifibranor for PPARα/δ/γ activation were low (Figure 1A(b,d)) and the maximal transactivation activities were rather partial (Figure 1A(c)); therefore, the lanifibranor used at the therapeutic doses could act as a partial PPAR pan agonist. In a recent network meta-analysis of interventions for NASH resolution without a worsening of fibrosis, semaglutide (a glucagon-like peptide-1 (GLP-1) receptor agonist/antidiabetic medication), pioglitazone (45 mg/day), and pioglitazone plus vitamin E were ranked as the most effective interventions with *P*-scores of 0.906, 0.890, and 0.826, respectively [52]. Vitamin E alone, lanifibranor, and pioglitazone (30 mg/day) were considered to be second class with *P*-scores of 0.706, 0.692, and 0.635, respectively [51]. Lanifibranor is considered to be superior to pioglitazone in that it can effectively alleviate fibrosis [52]. However, the efficacies of lanifibranor for PPARγ activation match those of pioglitazone; and therefore, PPARγ-related potent adverse effects should be carefully monitored during lanifibranor treatment.

The next promising drug may be saroglitazar, the PPARα/γ dual agonist. Our recent structural studies revealed that the carboxylic acid of saroglitazar was attached to the four consensus amino acids in both PPARα/γ-LBDs, although the orientation of its phenylpyrrole moiety was rotated 121.8 degrees to accommodate for PPARα/γ-LBDs but not PPARδ-LBD (Figure 1C(a)) [12]. Saroglitazar has been used for treating diabetic dyslipidemia in India [53], and thus its safety is fundamentally proven. A recent meta-analysis of ten clinical studies demonstrated that the treatment with saroglitazar (4 mg/day; either monotherapy or in combination) could significantly improve liver enzyme levels, reduce liver stiffness, and improve metabolic parameters (serum glucose and lipid profile) in patients with NAFLD or NASH [54]. Another recent network meta-analysis of 22 randomized controlled studies revealed that saroglitazar was significantly superior to the GLP-1 receptor agonists, including semaglutide, in improving ALT levels [55]. Overall, saroglitazar may be equal to or more effective than semaglutide for treating NASH.

We accept that safety-proven bezafibrate could be a third-class candidate. Bezafibrate belongs to clinically used fibrate-class PPARα agonists but does exhibit PPAR pan activities (Figure 2D(a–e)) [10] and has only been tested so far in clinical trials for anticancer drug-induced NASH [56,57]. Several animal experiments have proven its effectiveness in treating NAFLD/NASH models [58,59,60,61], and therefore, the repositioning of the lipid-lowering bezafibrate to treat NASH is anticipated. Pemafibrate could be another third-class candidate because of several recent reports that demonstrated that pemafibrate improved the FibroScan–aspartate aminotransferase (FAST) scores (a novel index of NASH conditions) [62,63,64] and similar scores in patients with NAFLD in several retrospective studies [65,66]. The high PPARα efficacy and selectivity of pemafibrate (at therapeutic doses) could enable combinatorial therapy with PPARδ/γ agonists for treating NASH.

PPAR agonists may also have therapeutic effects on cirrhosis. Lanifibranor (100 mg/kg/day, p.o.) ameliorated fibrosis and portal hypertension in rat models of decompensated cirrhosis [67]. Saroglitazar (4 mg/day) was effective and safe in improving biochemical and elastography parameters in patients with compensated cirrhosis [68]. Such PPAR agonists may be a new treatment option in the stage of cirrhosis where existing options are limited. Furthermore, treatment with PPAR agonists has been shown to prevent HCC in animal models by reducing NASH progression; saroglitazar (3 mg/kg/day, p.o.) completely prevented hepatic tumorigenesis [69] and pioglitazone inhibited the progression of hepatic steatosis and fibrosis, and reduced preneoplastic lesions [70]. NASH and alcohol had the fastest-growing age-standardized death rates between 2010 and 2019 [71]; and therefore, PPAR agonists may effectively reduce NASH-related mortality.

NAFLD/NASH is a multisystem disease with effects beyond the liver that increase the risk of incident T2DM, cardiovascular disease, heart failure, chronic kidney disease, and extra-hepatic cancers [72,73,74,75]. The future discovery of anti-NASH (or anti-metabolic disease) PPAR agonists will unequivocally have fundamental benefits from the in-depth functional and structural investigation of PPARα/δ/γ-LBD–ligand molecular interactions. Lanifibranor, saroglitazar, bezafibrate, and pemafibrate can contribute as immediate anti-NASH therapeutics or be lead compounds of PPAR selective agonists or pan agonists for various metabolic diseases.

## Figures and Tables

**Table 1:** PPAR Agonists in Completed or Terminated Clinical Trials against NAFLD/NASH (as of 2023/8/14).

Drug	NCT Number	Phase	Status	Participants	End Date	Liver Histology					Non-Invasive Tests		Blood Tests	
						Steatosis	Ballooning	Inflammation	Fibrosis	Total Score	Fat Content	Stiffness	ALT	AST
PPARα/δ/γ pan agonist														
Lanifibranor	NCT03008070 (https://classic.clinicaltrials.gov/ct2/show/NCT03008070)	2	Completed	247	2020/3/16	↓	↓	NS	↓	↓			↓	↓
PPARα/δ dual agonist														
Elafibranor	NCT01694849 (https://classic.clinicaltrials.gov/ct2/show/NCT01694849)	2	Completed	275	2015/12	↓	↓	NS	↓	↓			↓	
	NCT02704403 (https://classic.clinicaltrials.gov/ct2/show/NCT02704403)	3	Terminated	2157	2020/10/28					NS				
	NCT03883607 (https://classic.clinicaltrials.gov/ct2/show/NCT03883607)	2	Terminated	10	2020/6/16	No description (pharmacokinetics, pharmacodynamics, safety, and tolerability data only)								
	NCT03953456 (https://classic.clinicaltrials.gov/ct2/show/NCT03953456)	2	Terminated	17	2020/7/14	No description								
PPARα/γ dual agonist														
Saroglitazar	NCT02265276 (https://classic.clinicaltrials.gov/ct2/show/NCT02265276)	3	Unknown	100	2015/9	No description								
	NCT03061721 (https://classic.clinicaltrials.gov/ct2/show/NCT03061721)	2	Completed	106	2020/12/15						↓	↓		
	NCT03863574 (https://classic.clinicaltrials.gov/ct2/show/NCT03863574)	2	Completed	16	2020/10/30	No description								
	NCT04193982 (https://classic.clinicaltrials.gov/ct2/show/NCT04193982)	3	Unknown	250	2021/10/31	No description								
PPARα agonists														
Fenofibrate (α/γ)	NCT00252499 (https://classic.clinicaltrials.gov/ct2/show/NCT00252499)	NA	Terminated	13	2010/8	No description (protocol drug change required new clinicaltrails.gov entry)								
	NCT00262964 (https://classic.clinicaltrials.gov/ct2/show/NCT00262964)	NA	Completed	51	2008/12	No description								
	NCT01289639 (https://classic.clinicaltrials.gov/ct2/show/NCT01289639)	NA	Terminated	11	2014/8	No description								
	NCT02354976 (https://classic.clinicaltrials.gov/ct2/show/NCT02354976)	2	Completed	78	2016/5/26						↑		NS	↑
	NCT02781584 (https://classic.clinicaltrials.gov/ct2/show/NCT02781584)	2	Completed	220	2020/12/17	No description (safety and tolerability data only)								
	NCT02891408 (https://classic.clinicaltrials.gov/ct2/show/NCT02891408)	1	Completed	74	2019/5/13	No description (pharmacokinetics data only)								
Pemafibrate	NCT03350165 (https://classic.clinicaltrials.gov/ct2/show/NCT03350165)	2	Completed	118	2020/6/30						NS	↓	↓	NS
PPARδ agonist														
Seladelpar	NCT03551522 (https://classic.clinicaltrials.gov/ct2/show/NCT03551522)	2	Terminated	181	2020/8/10	No description								
PPARγ agonists														
Pioglitazone	NCT00013598 (https://classic.clinicaltrials.gov/ct2/show/NCT00013598)	2	Completed	30	2004/3	No description								
	NCT00062764 (https://classic.clinicaltrials.gov/ct2/show/NCT00062764)	2	Completed	18	2009/2	No description								
	NCT00063622 (https://classic.clinicaltrials.gov/ct2/show/NCT00063622)	3	Completed	247	2009/9	↓	↓	↓	NS				↓	↓
	NCT00227110 (https://classic.clinicaltrials.gov/ct2/show/NCT00227110)	4	Completed	55	2006/1	↓	↓	↓	NS		↓		↓	↓
	NCT00441272 (https://classic.clinicaltrials.gov/ct2/show/NCT00441272)	2	Completed	100	NP	No description								
	NCT00633282 (https://classic.clinicaltrials.gov/ct2/show/NCT00633282)	2	Completed	184	2011/8	No description (only in combination with lifestyle intervention; PMID: 26252777)								
	NCT00994682 (https://classic.clinicaltrials.gov/ct2/show/NCT00994682)	4	Completed	176	2014/12	↓	↓	↓	NS	↓	↓		↓	↓
	NCT01289639 (https://classic.clinicaltrials.gov/ct2/show/NCT01289639)	NA	Terminated	11	2014/8	No description (only baseline data published; PMID: 24360972, 24740208)								
	NCT01002547 (https://classic.clinicaltrials.gov/ct2/show/NCT01002547)	4	Completed	105	2016/12/31	No description (only in combination with vitamin E; PMID: 31332029)								
	NCT01068444 (https://classic.clinicaltrials.gov/ct2/show/NCT01068444)	2	Completed	90	2020/7	↓	NS	↓	NS	↓	↓		↓	↓
	NCT01431521 (https://classic.clinicaltrials.gov/ct2/show/NCT01431521)	1	Completed	31	2012/10/1	No description								
	NCT01703260 (https://classic.clinicaltrials.gov/ct2/show/NCT01703260)	2	Terminated	20	2014/9	No description								
	NCT02265276 (https://classic.clinicaltrials.gov/ct2/show/NCT02265276)	3	Unknown	100	2015/9	No description								
	NCT02365233 (https://classic.clinicaltrials.gov/ct2/show/NCT02365233)	4	Terminated	5	2016/12/31	No description								
	NCT02875821 (https://classic.clinicaltrials.gov/ct2/show/NCT02875821)	4	Completed	44	2017/6/7	No description								
	NCT03646292 (https://classic.clinicaltrials.gov/ct2/show/NCT03646292)	4	Unknown	60	2021/2	No description								
	NCT03796975 (https://classic.clinicaltrials.gov/ct2/show/NCT03796975)	4	Completed	120	2019/11/20	No description								
	NCT03910361 (https://classic.clinicaltrials.gov/ct2/show/NCT03910361)	4	Completed	51	2020/7/2	No description								
	NCT03950505 (https://classic.clinicaltrials.gov/ct2/show/NCT03950505)	4	Unknown	60	2020/12	No description								
	NCT05521633 (https://classic.clinicaltrials.gov/ct2/show/NCT05521633)	3	Completed	96	2022/5/24	No description								
Rosiglitazone	NCT00252499 (https://classic.clinicaltrials.gov/ct2/show/NCT00252499)	NA	Terminated	13	2010/8	No description (protocol drug change required new clinicaltrails.gov entry)								
	NCT00492700 (https://classic.clinicaltrials.gov/ct2/show/NCT00492700)	2	Completed	63	NP	↓	NS	NS	NS	NS			↓	↓
	NCT00699036 (https://classic.clinicaltrials.gov/ct2/show/NCT00699036)	2	Unknown	165	2009/8	No description								
	NCT01406704 (https://classic.clinicaltrials.gov/ct2/show/NCT01406704)	4	Terminated	26	2013/12	No description								
Lobeglitazone	NCT02285205 (https://classic.clinicaltrials.gov/ct2/show/NCT02285205)	4	Completed	38	2015/11						↓	NS	↓	↓

NCT, US National Library of Medicine Clinical Trials; NA, not applicable; NP, not provided; NS, not significant; ↑, up-regulated (exacerbated); ↓, down-regulated (alleviated).

**Table 2:** PPAR Agonists in Ongoing Clinical Trials against NAFLD/NASH (as of 2023/8/14).

Drug	NCT Number	Phase	Status	Participants	Subject	Start Date	ESC Date
PPARα/δ/γ pan agonists							
Chiglitazar	NCT05193916 (https://classic.clinicaltrials.gov/ct2/show/NCT05193916)	2	Recruiting	100	NASH with elevated triglyceride and insulin resistance	2022/3/21	(2023/11)
Lanifibranor	NCT03459079 (https://classic.clinicaltrials.gov/ct2/show/NCT03459079)	2	Recruiting	54	T2DM and NAFLD	2018/8/14	(2024/4/14)
	NCT04849728 (https://classic.clinicaltrials.gov/ct2/show/NCT04849728)	3	Recruiting	1000	NASH with F2/F3 stage of liver fibrosis	2021/8/19	(2026/9/30)
	NCT05232071 (https://classic.clinicaltrials.gov/ct2/show/NCT05232071)	2	Recruiting	63	T2DM and NASH	2022/6/29	(2023/12/31)
PPARα/γ dual agonist							
Saroglitazar	NCT03617263 (https://classic.clinicaltrials.gov/ct2/show/NCT03617263)	2	Recruiting	90	NAFLD in women with polycystic ovarian syndrome	2018/12/4	(2024/7)
	NCT03639623 (https://classic.clinicaltrials.gov/ct2/show/NCT03639623)	2	Recruiting	15	Liver transplant recipients with NAFLD	2019/2/25	2023/6
	NCT04469920 (https://classic.clinicaltrials.gov/ct2/show/NCT04469920)	1	Recruiting	100	NASH with advanced fibrosis	2020/7/16	(2024/10)
	NCT05011305 (https://classic.clinicaltrials.gov/ct2/show/NCT05011305)	2	Recruiting	240	NASH	2021/8/18	(2023/12)
	NCT05211284 (https://classic.clinicaltrials.gov/ct2/show/NCT05211284)	2	Recruiting	160	NASH with human immunodeficiency virus	2022/9/26	(2025/3/1)
	NCT05872269 (https://classic.clinicaltrials.gov/ct2/show/NCT05872269)	4	Not yet	1500	NAFLD with comorbidities (obesity, T2DM, dyslipidemia, or metabolic syndrome)	2023/7/20	(2025/6/10)
PPARγ agonist							
Pioglitazone	NCT04501406 (https://classic.clinicaltrials.gov/ct2/show/NCT04501406)	2	Recruiting	166	NASH in T2DM	2020/12/15	(2027/8/31)
	NCT04976283 (https://classic.clinicaltrials.gov/ct2/show/NCT04976283)	4	Recruiting	123	Liver fat in T2DM and NAFLD	2021/9/15	(2023/11/15)
	NCT05254626 (https://classic.clinicaltrials.gov/ct2/show/NCT05254626)	4	Recruiting	100	NASH	2022/8/1	(2025/8)
	NCT05305287 (https://classic.clinicaltrials.gov/ct2/show/NCT05305287)	4	Recruiting	60	NAFLD in T2DM	2022/11/1	(2027/3/31)
	NCT05422092 (https://classic.clinicaltrials.gov/ct2/show/NCT05422092)	NA	Not yet	80	NAFLD in T2DM	2022/9/20	(2023/12)
	NCT05513729 (https://classic.clinicaltrials.gov/ct2/show/NCT05513729)	NA	Recruiting	80	NAFLD in T2DM	2022/8/18	(2024/3/1)
	NCT05605158 (https://classic.clinicaltrials.gov/ct2/show/NCT05605158)	3	Not yet	56	NASH	2022/11	(2024/11)
	NCT05813249 (https://classic.clinicaltrials.gov/ct2/show/NCT05813249)	4	Recruiting	180	NAFLD in obesity and/or T2DM	2023/2/15	(2024/8/15)
	NCT05942963 (https://classic.clinicaltrials.gov/ct2/show/NCT05942963)	4	Not yet	240	NAFLD and T2DM	(2023/10)	(2024/4)

NCT, US National Library of Medicine Clinical Trials; NA, not applicable; ESC, estimated study completion date with future date in parentheses.
